# Hydrogen Peroxide Stimulates Activity and Alters Behavior in *Drosophila melanogaster*

**DOI:** 10.1371/journal.pone.0007580

**Published:** 2009-10-28

**Authors:** Dhruv Grover, Daniel Ford, Christopher Brown, Nicholas Hoe, Aysen Erdem, Simon Tavaré, John Tower

**Affiliations:** Molecular and Computational Biology Program, Department of Biological Sciences, University of Southern California, Los Angeles, California, United States of America; UCLA, United States of America

## Abstract

Circadian rhythms in animals are regulated at the level of individual cells and by systemic signaling to coordinate the activities of multiple tissues. The circadian pacemakers have several physiological outputs, including daily locomotor rhythms. Several redox-active compounds have been found to function in regulation of circadian rhythms in cells, however, how particular compounds might be involved in regulating specific animal behaviors remains largely unknown. Here the effects of hydrogen peroxide on *Drosophila* movement were analyzed using a recently developed three-dimensional real-time multiple fly tracking assay. Both hydrogen peroxide feeding and direct injection of hydrogen peroxide caused increased adult fly locomotor activity. Continuous treatment with hydrogen peroxide also suppressed daily locomotor rhythms. Conditional over-expression of the hydrogen peroxide-producing enzyme superoxide dismutase (SOD) also increased fly activity and altered the patterns of locomotor activity across days and weeks. The real-time fly tracking system allowed for detailed analysis of the effects of these manipulations on behavior. For example, both hydrogen peroxide feeding and SOD over-expression increased all fly motion parameters, however, hydrogen peroxide feeding caused relatively more erratic movement, whereas SOD over-expression produced relatively faster-moving flies. Taken together, the data demonstrate that hydrogen peroxide has dramatic effects on fly movement and daily locomotor rhythms, and implicate hydrogen peroxide in the normal control of these processes.

## Introduction

*Drosophila melanogaster* exhibits numerous complex behaviors, including walking, flight, grooming [1], foraging [2], fighting [3], mating [4], [5] and egg-laying [6], and most of these behaviors are under circadian control. The central circadian pacemakers in the mammalian and fly brains involve cellular feedback loops regulated at the level of protein modification and turnover, transcription and translation, and can coordinate biological rhythms throughout the animal in response to stimuli such as heat and light [7], [8]. The mechanisms for the coordination of rhythms in multiple tissues are unknown, however in mammals circulating hormones such as glucocorticoids have been implicated.

Cell autonomous oscillators have been characterized in both yeast and mammalian cells [9]. The yeast oscillator regulates the expression of both metabolism and detoxification (Phase I/II response-like) genes, and creates a metabolic cycle consisting of distinct oxidative and reductive periods. This temporal separation of potentially antagonistic biochemical pathways may optimize cell function and repair processes [10]. These results extend to metazoans, where the central pacemaker and tissue pacemakers control circadian expression of similar metabolism and detoxification gene sets, as well as additional genes such as those of the innate immune response [11], [12]. Strikingly, these same gene sets are altered during aging [13], [14] and in response to aging interventions across species [15], [16], supporting a link between circadian rhythms, metabolism/detoxification cycles and life span regulation. Consistent with this link, both aging and the oxidative stressor paraquat have been shown to alter *Drosophila* sleep cycles [17], [18], and the toxic effects of sleep deprivation can be ameliorated by certain heat shock proteins (hsps) [19], which are in turn induced in response to oxidative stress and aging [14], [20]–[22]. In mice, when the circadian rhythm genes *Period 1* and *Period 2* were simultaneously knocked-out, in addition to disrupted rhythms, the animals displayed signs of premature aging, decreased ability to repair DNA damage, and an increase in the incidence of tumors [23] – all phenotypes associated with oxidative stress.

In addition to circadian pacemakers regulating metabolism, several mechanisms have been defined through which metabolism can in turn regulate circadian rhythms [24]. For example, in mammals, the NAD(P)/NAD(P)H ratio regulates clock proteins via conserved PAS domains. PAS domain proteins can also be regulated by additional redox-active compounds, including Heme, the Heme breakdown product CO gas, as well as NO gas.

One of the major stumbling blocks for detailed analysis of the behavioral effects of genetic and pharmacological manipulations in *Drosophila* has been the lack of methods capable of tracking flies and quantifying their behavior. To this end, several machine vision tracking systems have been developed recently to analyze behaviors such as walking movements [25]–[28] and flight trajectories in single [29], [30] and multiple [31] *Drosophila* flies in 2D. We have recently developed a tracking method involving multiple video cameras that allows for detailed analysis of the movement of groups of flies through 3D space in real-time [32], along with simultaneous assay of transgenic reporter constructs expressing GFP or DsRED [22], [33]. These methods provide an ideal way to analyze the effects of chemical and transgenic manipulations on fly behavior and rhythms. Hydrogen peroxide is a good candidate for a behavior regulator, as it is the most stable and diffusible of reactive oxygen species (ROS), and has been shown to function as a cellular signaling molecule in several other processes [34]. Here hydrogen peroxide was found to stimulate fly motion parameters and to alter daily locomotor rhythms, consistent with a normal role for hydrogen peroxide in the regulation of fly movement and behavior.

## Results

**Figure 1:**
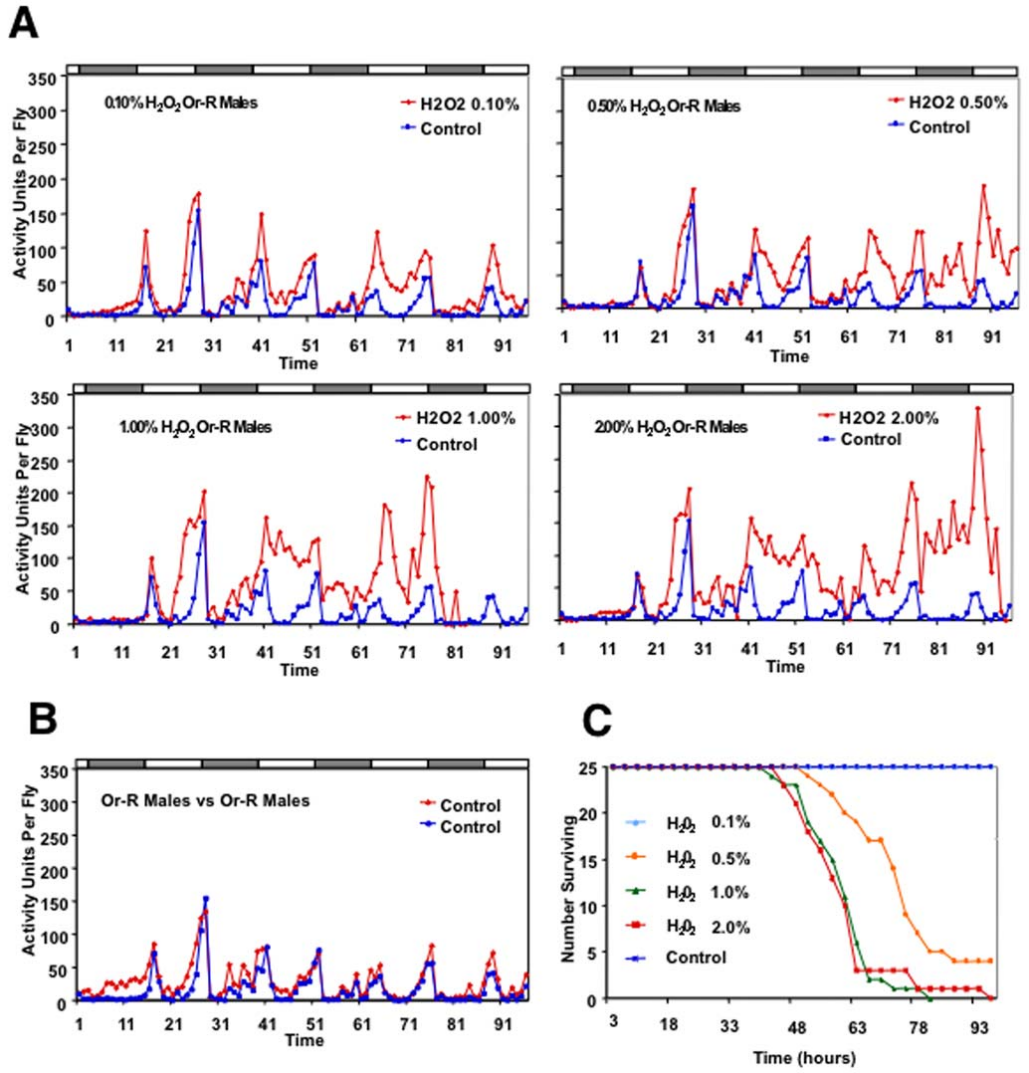
Effect of dietary hydrogen peroxide on adult fly activity. (A, B) Average activity of 25 young adult male flies was measured using the DAM. Alternating light and dark boxes above the charts indicate the 12 hr-light/12 hr-dark cycle in which the flies were housed during the experiment. (A) The dose-response for flies fed hydrogen peroxide (H_2_O_2_) is presented with data expressed as activity units per fly; the control data is the same in each panel. Drug treatment began at 21 hours. (B) Oregon-R wild-type male flies in one DAM (red) compared to a duplicate vial of flies in a second DAM (blue). (C) Survival curves for flies fed increasing amounts of hydrogen peroxide, and mock-fed controls. All experiments were repeated with similar results.

The average activity of groups of 25 flies was measured using the *Drosophila* Activity Monitor (DAM) system (Trikinetics), in which three rings of infrared beams record fly movement in a small chamber. When hydrogen peroxide was fed to flies it increased activity in a dose-dependent manner across a period of several days (Figure 1A; Supplemental Figure S1; Table 1). Concentrations greater than 0.1% hydrogen peroxide also caused significant mortality (Figure 1C). One way that cells control ROS levels is with abundant cytoplasmic catalase enzyme that converts excess hydrogen peroxide to water and oxygen. Tertiary butyl hydroperoxide (TBHP) has some similarity in chemical properties with hydrogen peroxide, but cannot be degraded by catalase [35]. When fed to flies TBHP also caused increases in activity, however it was also more toxic (data not shown). At low concentrations TBHP appeared to suppress the normal diurnal activity pattern, such that flies were more active than controls during the sleep periods, and less active than controls during the active periods (Supplemental Figure S2). Treatment of flies with the catalase enzyme inhibitor 3-amino-1,2,4-triazol (AMT) [36] was toxic without causing detectable increase in fly activity (data not shown).

**Table 1:** Activity statistics for flies fed hydrogen peroxide.

Dose	ar1	ma1	sar1	sma1	mean	Z-statistic	p-Value
**0.1% H_2_O_2_**	0.8455 [0.1224]	−0.0263 [0.1636]	−0.1898 [0.4203]	−0.5924 [0.7413]	36.1838 [1.6712]	**7.64**	**<<0.001**
**0.5% H_2_O_2_**	0.6833 [0.1089]	0.0556 [0.1423]	−0.3814 [0.1465]	−0.9704 [0.3010]	43.6421 [2.3048]	**8.92**	**<<0.001**
**1.0% H_2_O_2_**	0.8298 [0.1233]	−0.0782 [0.1716]	−0.3866 [0.2141]	−0.3241 [0.2618]	71.0559 [1.6092]	**25.23**	**<<0.001**
**2.0% H_2_O_2_**	0.8742 [0.0141]	−0.1393 [0.0140]	−0.7092 [0.0070]	0.2054 [0.0134]	78.8187 [2.4154]	**21.65**	**<<0.001**
**Control**	0.3874 [0.1206]	0.1655 [0.2468]	−0.2852 [0.1644]	−0.9780 [0.2394]	20.4755 [1.1958]		

Average activity statistics for each set of flies given a dose of 0.1% H_2_O_2_, 0.5% H_2_O_2_, 1.0% H_2_O_2_ and 2.0% H_2_O_2_, and Controls, respectively. The data were modeled using an ARIMA time series approach (Methods). The model selected was ARIMA ((1, 0, 1)x(1, 1, 1)_24_). The coefficients listed below are ar1, ma1, seasonal ar1, seasonal ma1, and mean with standard errors below in parentheses. The statistic 
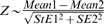
 is presented, as well as p-values for tests of the significance of difference of the means between experimental and control.

**Figure 2:**
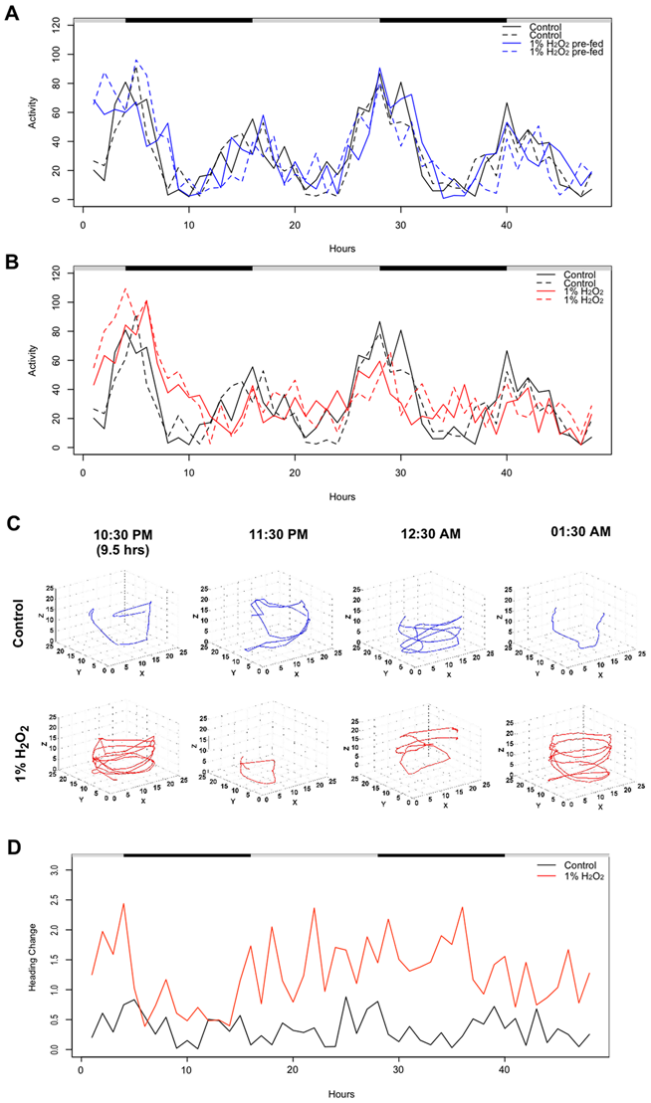
Video tracking assay of effects of dietary hydrogen peroxide on fly activity and behavior. Multiple Oregon-R wild-type male flies, 5 days old, were placed in two vials and tracked simultaneously for 48 hours. The first vial contained four flies on normal food, two of which had been pre-fed 1.0% hydrogen peroxide (H_2_O_2_) for two hours before being transferred to the vial. The second vial contained two flies and food supplemented to 1.0% hydrogen peroxide. Black/white bars indicate the light/dark cycle in which the flies were housed. Activity is expressed as distance moved (cm) per hour. (A) Two control flies (black lines, one solid and one dashed) and two flies pre-fed 1.0% H_2_O_2_ (blue lines, one solid and one dashed). B. Two control flies (black lines, one solid and one dashed) and two flies continuously fed 1.0% H_2_O_2_ (red lines, one solid and one dashed). The control fly data is the same in (A) and (B). C. The 3D tracks of individual male flies were recorded for two-minute periods at one-hour intervals beginning 9.5 hours after first exposure to control food or food adjusted to 1.0% H_2_O_2_. Statistical analysis is presented in Table 2B. D. Effect of dietary hydrogen peroxide on fly heading changes. A control fly and a fly fed continuously with 1% hydrogen peroxide were tracked for 48 hours using the video tracking assay, and average heading changes per hour are presented. The data were modeled using ARIMA(5,0,0). Control mean = 0.3396, SEM = 0.0223. 1% H_2_O_2_ mean = 1.2836, SEM = 0.1359. Z statistic = 6.8546, p<<0.001 (highly significant). All experiments were repeated with similar results (data not shown).

One limitation of the DAM system is that it measures average activity in discrete time intervals, and offers little information regarding the specific nature of the activity. In order to better study *Drosophila* movement behavior and the effects of hydrogen peroxide on it, the automated video tracking system was applied [22], [32], [33]. Multiple flies were detected and tracked simultaneously using an array of calibrated and synchronized digital video cameras operating at 60 frames per second, allowing for real-time analysis of fly movement. The resulting fly trajectories are typically cylindrical (for example, see Figure 2C), corresponding to the round shape of the vials in which the flies were housed. *Drosophila* flies tend to favor the periphery of their container space, a behavior termed “centrophobism” [22], [25], [28].

**Table 2:** Behavioral statistics for comparison of flies with dietary and transgenic manipulations.

			Speed	Average Speed	Heading	Average Heading
**A. Control vs 1% H_2_O_2_**	Time Point 1	Control	0.55 [0.047]	0.54 [0.046]	0.0000044 [0.00000030]	0.000017 [0.0000010]
		Experimental	13.42 [1.55]	12.96 [0.87]	0.000050 [0.0000040]	0.000066 [0.0000050]
	Time Point 2	Control	2.25 [0.25]	2.07 [0.16]	0.000065 [0.0000080]	0.00019 [0.000020]
		Experimental	9.23 [1.11]	9.09 [0.65]	0.00041 [0.000030]	0.00052 [0.000040]
	Time Point 3	Control	0.47 [0.021]	0.47 [0.021]	0.000008 [0.00000050]	0.000032 [0.0000020]
		Experimental	7.54 [0.66]	7.38 [0.46]	0.14 [0.0060]	0.16 [0.0062]
	**Increase**		**9.23**	**9.56**	**1814.73**	**671.91**
**B. Control vs 1% H_2_O_2_ (First time point is at the 9.5 hour mark)**	10:30PM	Control	0.0041 [0.00022]	0.0040 [0.00019]	0.00095 [0.000066]	0.0037 [0.00024]
		Experimental	0.014 [0.00099]	0.018 [0.0011]	0.0058 [0.00053]	0.021 [0.0012]
	11:30PM	Control	0.035 [0.0041]	0.035 [0.0033]	0.12 [0.018]	0.37 [0.041]
		Experimental	0.0091 [0.00075]	0.0097 [0.00062]	0.026 [0.0071]	0.081 [0.014]
	12:30AM	Control	0.016 [0.0012]	0.015 [0.0010]	0.00031 [0.000019]	0.0012 [0.000069]
		Experimental	0.0041 [0.00035]	0.0048 [0.00048]	0.000073 [0.0000093]	0.00022 [0.000020]
	1:30AM	Control	0.0049 [0.00016]	0.0049 [0.00017]	0.0000048 [0.00000024]	0.000019 [0.00000094]
		Experimental	0.0092 [0.00057]	0.010 [0.00056]	0.000037 [0.0000021]	0.00013 [0.0000073]
**C. MnSOD(2)12, +DOX vs -DOX**	Time Point 1	Control	0.20 [0.023]	0.19 [0.019]	0.00011 [0.000014]	0.00017 [0.000022]
		Experimental	0.47 [0.038]	0.44 [0.031]	0.00097 [0.00011]	0.0021 [0.00025]
	Time Point 2	Control	0.052 [0.0033]	0.52 [0.0021]	0.00030 [0.000014]	0.00031 [0.000013]
		Experimental	1.66 [0.18]	2.19 [0.13]	0.00043 [0.000053]	0.0011 [0.000065]
	Time Point 3	Control	0.044 [0.0028]	0.044 [0.0017]	0.00094 [0.000053]	0.0010 [0.000052]
		Experimental	17.23 [2.65]	9.39 [2.78]	0.0018 [0.00023]	0.0042 [0.00032]
	Time Point 4	Control	0.022 [0.0017]	0.022 [0.0012]	0.000019 [0.0000012]	0.000025 [0.0000013]
		Experimental	3.57 [1.28]	3.58 [0.85]	0.00014 [0.000012]	0.00023 [0.000021]
	**Increase**		**72.11**	**20.10**	**2.44**	**5.069**

Five flies were used in each experiment. Numbers presented are averages over 2 minutes of tracking observations at 60 frames/sec with standard errors below in parentheses. Increase indicates the ratio of activity of experimental and control flies. A. Oregon-R wild type male flies fed 1.0% H_2_O_2_ and controls, assayed at three-hour intervals after contact with drug for one hour. B. Time interval where suppression of diurnal rhythms by H_2_O_2_ resulted in experimental flies that were less active than control. The first time point is 9.5 hours after the flies were exposed to H_2_O_2_. C. Flies with *MnSOD(2)12* over-expression (+DOX) and (-DOX) controls, assayed at three-hour intervals.

The real-time 3D tracking assay was used to determine how fly movement and trajectories might be affected by hydrogen peroxide treatment. The four parameters measured were speed, average speed, heading, and average heading (see Materials and methods). Average values and their corresponding standard errors for multiple time points after hydrogen peroxide administration are presented (Table 2A). Flies exhibited an increase in activity beginning on average approximately 11 minutes after first hydrogen peroxide exposure (Figure S3; Materials and methods). These changes included an approximately 9-fold increase in speed (flying and walking) over time (Table 2A). Additionally, it was observed that the flight path trajectories of treated flies were considerably more erratic, as evidenced by greater heading and average heading values (Table 2A; Figure 2D). This erratic movement is apparent in the more jagged appearance of the trajectories for hydrogen peroxide-fed flies relative to controls (Figure 2C).

Strikingly, while hydrogen peroxide increased fly activity on average, continuous administration of hydrogen peroxide was found to suppress daily locomotor rhythms. Feeding flies with 1% hydrogen peroxide for two hours caused an increase in activity, as indicated by increased distance moved per hour (Figure 2A). The increased activity returned to normal levels within a few hours, and subsequent daily locomotor rhythms were normal. However, when flies were continuously fed hydrogen peroxide, activity was initially increased, and then after about 12 hours the daily locomotor rhythms became suppressed: flies were more active than controls during the sleep periods, and less active than controls during the wake periods (Figure 2B,C). Even during the time intervals when flies fed hydrogen peroxide were less active than controls, based on distance moved per hour, their behavior was abnormal, in that movement was more erratic (greater heading changes) than a control fly moving at a comparable speed (Table 2B; Figure 2D). Because the activity measured by the DAM is a combination of distance moved and amount of heading changes, distinguishing between these parameters was only possible using the video tracking assay.

### Hydrogen Peroxide Injection and SOD Over-Expression Also Cause Increased Fly Activity

As described above, feeding flies hydrogen peroxide caused an increase in activity and alterations in behavior, beginning approximately 11 minutes after first exposure. One possibility is that after the fly ingests the hydrogen peroxide, it passes into the fly's circulatory system, and then causes chemical changes in cells that regulate behavior. Alternatively, the hydrogen peroxide might cause chemical changes in the cells that line the digestive tract, and this signal is then transduced to cells that regulate behavior via some signal other than hydrogen peroxide. Finally, it is conceivable that the fly senses hydrogen peroxide in its external environment, and this information is then transmitted to cells regulating behavior. To demonstrate that the behavioral changes were due to redox alterations taking place inside the fly's tissues and cells, two additional approaches were used: injection and transgenic manipulation.

**Figure 3:**
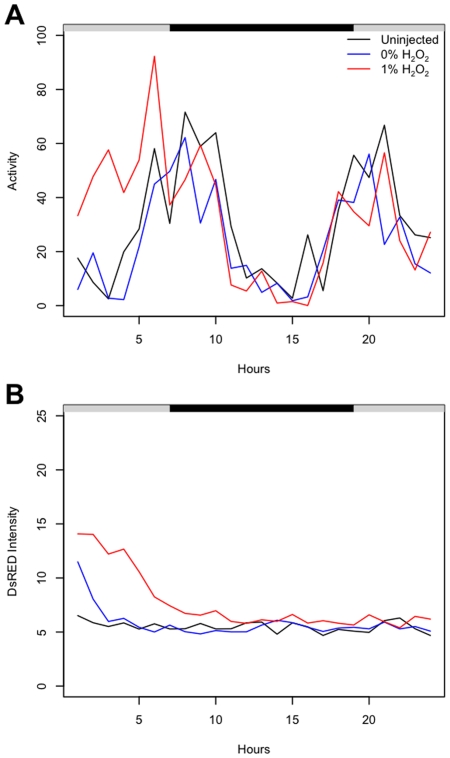
Effect of injecting hydrogen peroxide on fly activity, behavior and gene expression. Three male flies, 10 days old, were placed in a vial and tracked simultaneously for 24 hours. The flies were transgenic for the *hsp22*-DsRED reporter, strain 1MI1. Black/white bars indicate the light/dark cycle the flies were cultured under prior to the beginning of the experiment. Activity is expressed as distance moved (cm) per hour (A), and DsRED fluorescence intensity is expressed as the average pixel intensity per hour (B). Uninjected (black line), buffer injected (blue line) and 1.0% hydrogen peroxide (H_2_O_2_) injected (red line). The experiment was conducted four times with similar results, and a representative trial is presented.

Flies were injected in the open circulatory system (hemocoel) of the abdomen with ∼0.05 ul of PBS adjusted to 1% hydrogen peroxide, or with PBS alone as a control. Hydrogen peroxide injection caused an immediate increase in fly activity that persisted for approximately 6 hours, before returning to control levels (Figure 3A). Injection of hydrogen peroxide also caused the transient induction of a transgenic reporter that is sensitive to oxidative stress [21], [33], the *hsp22* gene promoter driving expression of DsRED (Figure 3B). Therefore, similar to the effect of dietary hydrogen peroxide, direct injection of hydrogen peroxide into the fly's circulatory system caused a reversible increase in fly activity.

In cells the superoxide dismutase (SOD) enzymes convert superoxide anions to hydrogen peroxide and oxygen. MnSOD is located exclusively in the inner mitochondrial space, whereas Cu/ZnSOD is located in the outer mitochondrial space and cytoplasm. Together these SOD enzymes are thought to be responsible for generating the majority of hydrogen peroxide in the cell [37]. A conditional transgenic system called “Tet-on” [38], [39] was used to cause tissue-general, doxycycline (DOX)-dependent over-expression of MnSOD or Cu/ZnSOD cDNAs in adult flies. Previously, over-expression of these cDNAs using the FLP-*out* system was shown to cause a proportional increase in enzyme activity [20], and similarly, both RNA and enzyme over-expression were confirmed for these new Tet-on MnSOD strains [16], [39] and Cu/ZnSOD strains (D. Ford and J. Tower, in preparation).

**Figure 4:**
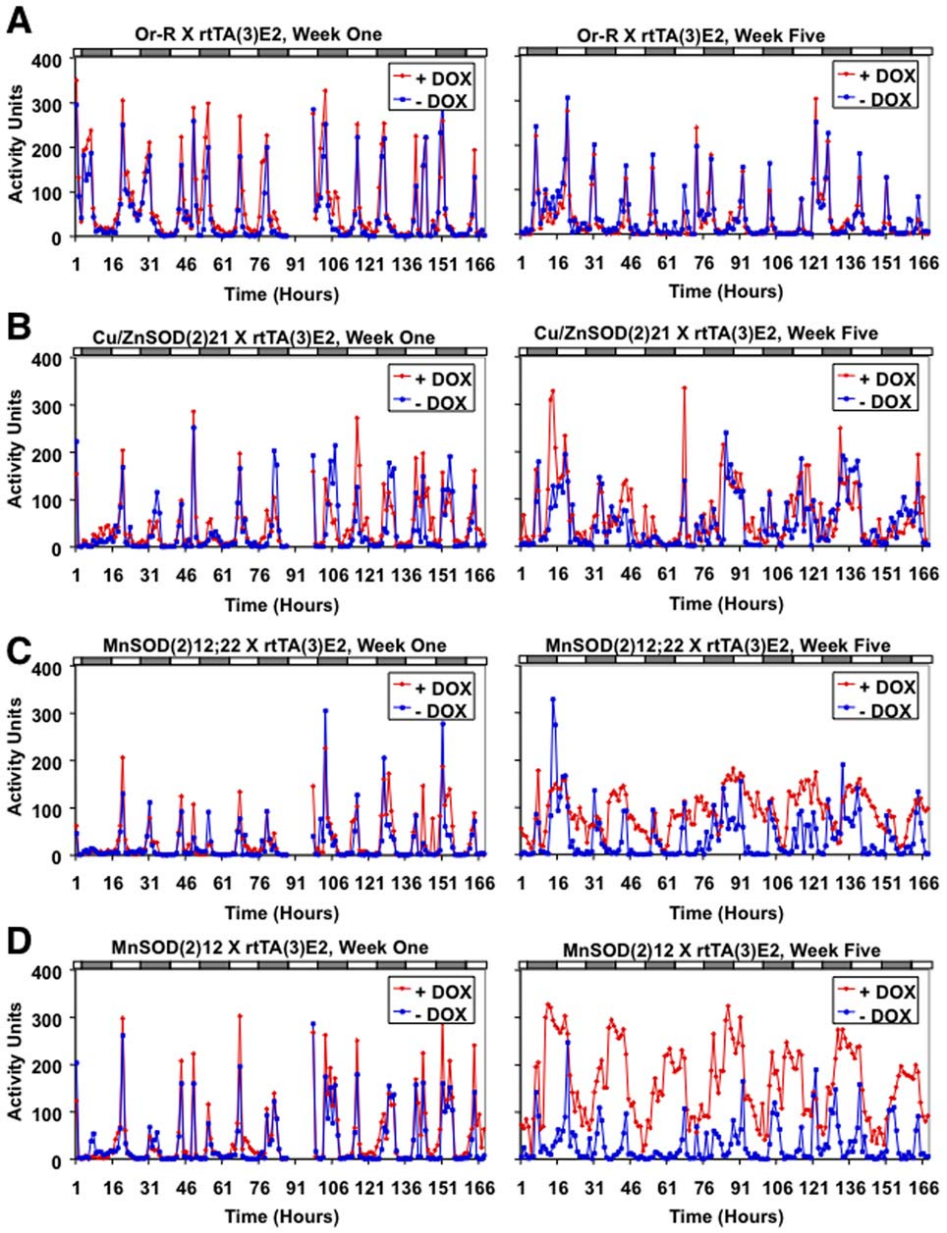
Effect of conditional over-expression of MnSOD or Cu/ZnSOD on average adult fly activity. The Tet-on transactivator insertion strain *rtTA(3)E2* was crossed to Or-R wild-type control flies to generate progeny containing only the driver (A), as well as to the indicated *Cu/ZnSOD* and *MnSOD* target construct strains to generate progeny containing both constructs (B–D). Young adult males (2–4 days old) were cultured on food ±DOX at 25 flies per vial, and assayed across five weeks of adult life span using the DAM. (A–D) The details of activity profiles across weeks one and five are presented, with control fly data (-DOX) indicated in blue, and experimental fly data (+DOX) indicated in red.

To investigate the effects of SOD over-expression on behavior and locomotor rhythms, activity was assayed using the DAM across five weeks of adult life span of control and experimental flies, in groups of 25 each. Both MnSOD and Cu/ZnSOD over-expression were found to cause a time-dependent increase in fly activity (Figure 4), consistent with a role for hydrogen peroxide. Larger average increases in activity per fly were observed upon over-expression of MnSOD than with Cu/ZnSOD (Table 3), consistent with an effect on behavior of hydrogen peroxide produced within the mitochondria.

**Table 3:** Activity statistics for flies with SOD over-expression over five weeks.

Genotype	Dose	ar1	ma1	sar1	sma1	mean	Z-statistic	p-Value
**Control**	**+DOX**	0.3141 [0.0386]	0.1187 [0.13330]	−0.1187 [0.0443]	−0.8260 [0.0307]	38.5524 [1.6639]	**−0.38**	**0.7**
	**−DOX**	0.6016 [0.0384]	−0.2758 [0.1150]	−0.0693 [0.0422]	−0.8695 [0.0263]	39.5620 [2.0596]		
***Cu/ZnSOD(2)21***	**+DOX**	0.4898 [0.0372]	−0.0596 [0.0907]	−0.1348 [0.0452]	−0.8091 [0.0330]	65.1372 [1.1280]	**9.89**	**<<0.001**
	**−DOX**	0.5896 [0.0395]	−0.2246 [0.1102]	−0.0812 [0.0434]	−0.8023 [0.0281]	41.8421 [2.0665]		
***MnSOD(2)12,22***	**+DOX**	0.7475 [0.0390]	−0.3192 [0.0783]	−0.0188 [0.0601]	−0.6259 [0.0523]	86.9012 [1.1039]	**30.36**	**<<0.001**
	**−DOX**	0.6983 [0.0357]	−0.3136 [0.0777]	−0.0397 [0.0491]	−0.7900 [0.0359]	40.2427 [1.0692]		
***MnSOD(2)12***	**+DOX**	0.7348 [0.0425]	−0.3621 [0.1046]	−0.1501 [0.0523]	−0.5957 [0.0478]	161.0170 [1.5933]	**54.52**	**<<0.001**
	**−DOX**	0.4112 [0.0359]	−0.1242 [0.1205]	−0.0035 [0.0500]	−0.8162 [0.0379]	39.5804 [1.5564]		

Average activity statistics for Control, *Cu/ZnSOD(2)21*, *MnSOD(2)12,22* and *MnSOD(2)12,* respectively. The DAM data were modeled using an ARIMA time series approach (Methods). The model selected was ARIMA ((1, 0, 1)x(1, 1, 1)_24_). The coefficients listed below are ar1, ma1, seasonal ar1, seasonal ma1, and mean with standard errors below in parentheses. The statistic 
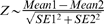
 is presented, as well as p-value results for tests of significance of difference of the means between +DOX and -DOX for each of the genotypes.

**Figure 5:**
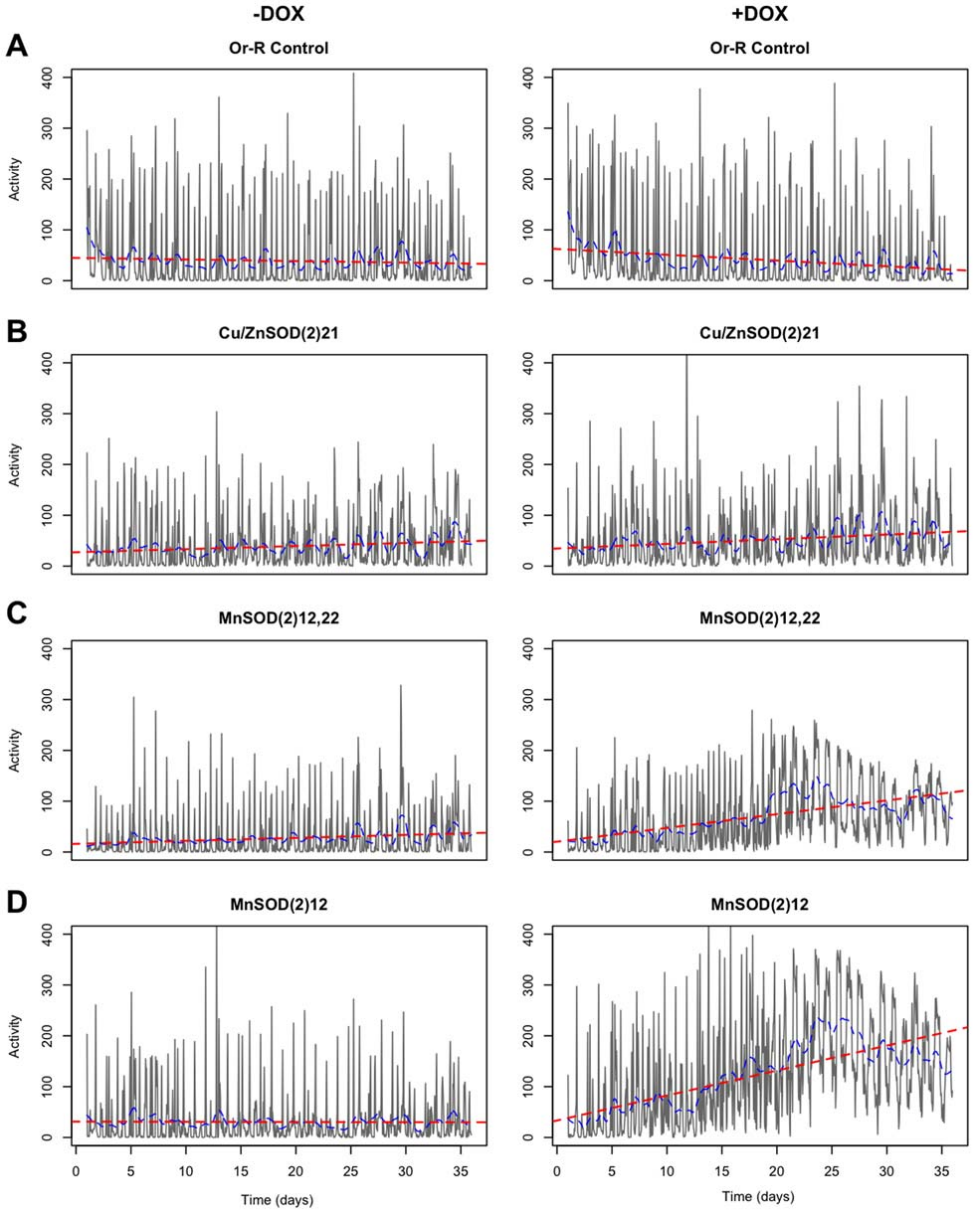
Activity trend of flies over-expressing SOD. Time series of average adult activity per fly across weeks one through five is presented for each set of flies, ±DOX, as indicated. A seasonal-trend decomposition procedure decomposed the raw time-series activity data (gray) into a “seasonal” component indicating rhythms, and a “trend” component indicative of changes in overall activity (blue). This was performed in R [63] using the function *stl*. A linear model was fit to the trend component of the raw activity time series (red) to demonstrate the trend of increasing activity for flies over-expressing SOD. Next, an ARIMA time series modeling approach (Material and methods) was utilized for further analysis, which revealed average activity values for -DOX and +DOX flies (with standard errors in parentheses). (A) Control -DOX 39.6 (2.05), +DOX 38.6 (1.66). (B) Progeny with *Cu/ZnSOD(2)21* -DOX, 41.8 (2.07), +DOX 65.1 (1.13). (C) Progeny with *MnSOD(2)12,22* -DOX 40.2 (1.07), +DOX 87 (1.10). (D) Progeny with *MnSOD(2)12* -DOX 39.6 (1.56), +DOX 161 (1.59).

To analyze the patterns of activity in flies with SOD over-expression, a seasonal-trend decomposition approach [40], as implemented in the R function *stl*, was applied to the average activity per fly data. This procedure decomposed the raw time-series activity data into a “seasonal” component indicating rhythms, and a “trend” component indicative of changes in overall activity. The rhythms of control flies were relatively constant across five weeks, although some gradual and subtle reductions in relative peak size and number per day were observed (Figure 5A). The activity trend of control flies (indicated by blue dashed line) dropped quickly from an initial high level, followed by several weeks of sustained activity. DOX treatment had no detectable effect on either rhythms or activity in control flies. In contrast, DOX treatment of SOD transgenic lines had dramatic effects on both the locomotor rhythms and the trend in activity in each case (Figure 5B–D; Table 3). SOD over-expression significantly increased activity, particularly in the fourth and fifth weeks. To a much lesser extent these effects were apparent even in the –DOX flies, perhaps indicating some leaky expression of the transgenes. Interestingly, while both dietary hydrogen peroxide and SOD over-expression greatly increased fly activity across all the movement parameters, there were some differences in effects. For example, hydrogen peroxide feeding produced a relatively more erratic flight path (greater heading and average heading values) while MnSOD over-expression caused relatively faster-moving flies (greater speed and average speed values) (Table 2A, 2C).

## Discussion

The real-time 3D tracking system allowed the analysis of specific effects of hydrogen peroxide and SOD over-expression on fly behavior and daily locomotor rhythms. Hydrogen peroxide was found to affect the activity, behavior and locomotor rhythms of *Drosophila*, suggesting a role for hydrogen peroxide in the normal regulation of these processes. Continuous dietary hydrogen peroxide stimulated several types of movement, while at the same time it tended to suppress daily locomotor rhythms. MnSOD over-expression stimulated activity in a similar way to hydrogen peroxide, causing flies to be more active and erratic. In flies with increased MnSOD expression, the patterns of daily locomotor rhythms appeared largely intact, but were significantly altered. The pattern of behavior across the day was altered in both the size and number of peaks, and in general activity was increased, particularly at later time points. In the MnSOD lines, at later time points activity was increased at all hours of the day to the extent that sleep was nearly eliminated. Surprisingly these hyperactive and sleep-deprived flies can have either reduced life span, as with line *MnSOD(2)12,22* [39], or increased life span, as found for line *MnSOD(2)12* [16].

Several previous observations are consistent with a link between SOD, hydrogen peroxide and behavior regulation. An activity monitor has been used to show that flies over-expressing catalase maintain activity for a longer period when challenged with toxic concentrations of hydrogen peroxide [41]. Melatonin has been identified as a circadian rhythm regulator in mammals, and has been reported to act as a scavenger of several different ROS [42], including hydrogen peroxide [43] (but see also [44]). Circadian variation in SOD enzyme activity is observed in primate erythrocytes [45]. Interestingly, genetic selection of mice for increased wheel running is associated with decreased MnSOD activity in liver tissue [46].

The fact that both dietary hydrogen peroxide and SOD over-expression had similar effects on behavior is consistent with the idea that these manipulations act through a similar mechanism, which most likely involves alterations in redox physiology in one or more critical tissues. The products of SOD enzyme are hydrogen peroxide and oxygen, however simply because SOD enzyme activity was increased it cannot be assumed that the phenotypic effects are due to increased hydrogen peroxide levels, as SOD could also conceivably act by altering superoxide levels, oxygen levels, metal scavenging or other mechanisms [47]. However, two lines of evidence point towards increased hydrogen peroxide as the relevant effector of SOD over-expression phenotypes: First is the similar effects of dietary hydrogen peroxide, injected hydrogen peroxide, and SOD over-expression on fly behaviors, as presented here; second is recent data from our research group indicating that SOD over-expression causes changes in gene expression that are highly similar to hydrogen peroxide feeding, including the preferential induction of genes bearing hydrogen peroxide response elements in their promoters ([16] and unpublished data).

One direct way to test this model would be to measure hydrogen peroxide levels in the cells of the SOD over-expressing flies. However, using simple spectrofluorometric assays we were unable to detect reproducible changes in hydrogen peroxide levels in whole-fly extracts, or from mitochondria isolated from SOD over-expressing flies (data not shown). This result is perhaps not surprising as it might be expected that changes in hydrogen peroxide levels sufficient to mediate signaling could be quite small and limited to within a narrow physiological range. Moreover, altered hydrogen peroxide signaling might occur only in a subset of fly cells, and might be limited to localized and transient changes within the relevant cells, and thus would not be apparent in whole-fly extracts. One possible approach for the future might be the use of redox-sensitive fluorescent proteins [48]–[52] as reporters in the control and SOD-over-expressing flies; however since so far no chemical or transgenic reporter has been shown to be completely specific for hydrogen peroxide this will not be a simple undertaking. Strikingly, it has recently been reported that mitochondria isolated from *Drosophila* heads show a circadian variation in the production of hydrogen peroxide [53], and in zebrafish larvae hydrogen peroxide has been shown to act as an systemic signaling molecule that recruits immune cells to sites of tissue damage [52]. These results are consistent with the idea that hydrogen peroxide may act as both an intracellular and intercellular signaling molecule.

Daily locomotor activity in *Drosophila* is regulated by the central circadian pacemaker, and it is possible that the effects of dietary hydrogen peroxide and SOD over-expression were caused by alterations in the circadian machinery. Several studies have demonstrated that the conserved circadian oscillator is regulated by cellular metabolism [9]. In particular, redox-active signaling molecules such as NADPH, CO, NO and heme have been shown to regulate circadian transcription factors such as Clock, Cycle and BMAL1 through conserved PAS protein domains [54], [55]. Since the mitochondria are a key regulator of metabolism and a primary source of cellular ROS, the data suggest that a retrograde redox signal from the mitochondria may normally be involved in regulating cellular oscillators and circadian rhythms. Hydrogen peroxide has not previously been directly implicated in these processes, however it is interesting to note that exogenous hydrogen peroxide has been shown to be capable of advancing the period of the yeast metabolic cycle [10]. The fact that MnSOD is a mitochondrial enzyme, that the mitochondria are the primary cellular source of hydrogen peroxide, and that dietary hydrogen peroxide and MnSOD over-expression both altered a direct output of circadian rhythms in flies (daily locomotor activity), suggests that hydrogen peroxide may be an additional retrograde signal affecting circadian rhythms. Aging is associated with increased oxidative stress and a deterioration of behaviors and circadian rhythms in both flies and humans. One possible model is that the oxidative stress associated with aging results in part from a breakdown in normal mitochondrial-nuclear signaling pathways involving periodic (circadian) variations in redox signaling molecules, including hydrogen peroxide [34], [56], [57].

## Materials and Methods

### *Drosophila* Strains and Culture

The wild-type Oregon-R *Drosophila melanogaster* strain was obtained from Bloomington *Drosophila* stock center. The flies were maintained on a standard cornmeal, yeast, dextrose, and agar medium at 25°C [39]. Age-synchronized cohorts of flies were collected over 48 hours from culture bottles or vials, and drug-treatment experiments used young (4–6 day old) Oregon-R male flies while experiments with transgenic flies used young (2–4 day old) male flies of the indicated genotypes. All reagents were purchased from Sigma-Aldrich. The Tet-on MnSOD and Cu/ZnSOD expression constructs were created by cloning the corresponding cDNAs [58] into the USC1.0 vector [59] and generating multiple independent transformant strains. Line *MnSOD(2)12* contains a single insert, while lines *MnSOD(2)12,22* and *Cu/ZnSOD(2)21* contain double inserts. These strains are additionally described and characterized elsewhere [16], [39] (D. Ford and J. Tower, in preparation). The *rtTA* driver construct consists of the rtTA transcription factor coding region downstream of the *actin5C* promoter, and the *rtTA(3)E2* insertion supports high-level, DOX dependent expression of target constructs in all tissues except the germ line [38]. For experiments involving SOD over-expression, flies of the desired genotypes were generated by crossing strains homozygous for the indicated *Cu/ZnSOD* and *MnSOD* transgene insertions to a strain containing the *rtTA(3)E2* driver to obtain progeny containing each construct. Control flies were generated by crossing the *rtTA(3)E2* driver strain to Oregon-R wild type flies to generate progeny containing only *rtTA(3)E2*.

### Drug Treatments

For the *Drosophila* Activity Monitor (DAM) experiments lasting 4 or 5 days (Figures 1, 2 and 4), control groups of flies received 1% sucrose in pure de-ionized H_2_O, while experimental groups received 1% sucrose solution plus the indicated concentration of H_2_O_2_ (0.1%, 0.5%, 1.0%, 2.0%), tertiary butyl hydroperoxide (TBHP) (0.05%, 0.1%), or 3-amino-1,2,4-triazol (AMT) (0.07%). For each solution 1.0mL was separately added to a *Drosophila* culture vial containing a single Kimwipe (Kimberly Clark) packed tightly at the bottom (∼10 mm thick layer) to absorb the solution. Professional standard polystyrene *Drosophila* culture vials (25×95 mm; Genesee Scientific) were used in all experiments. For the three-camera tracking assays, vials containing standard fly media stained with blue food coloring (Kroger brand) were used, as the blue colored background was required for efficient tracking. The food in the vial was adjusted to 1.0% H_2_O_2_ using a 30% H_2_O_2_ solution. The experiment began after the solution was given twenty-four hours to absorb into the media. For DAM experiments lasting 5 weeks (Figure 4), vials containing *Drosophila* media were adjusted to 64 µg/ml doxycycline (DOX) plus 64 µg/ml ampicillin, while control vials were adjusted 64 µg/ml ampicillin alone. Chemical adjustment of food was done by applying 100 µl of a 10X stock solution to the surface of food vials, which penetrates 1 ml of food after 48 hours incubation based on colored dye absorption. The ampicillin was present to help prevent any bacterial growth in the vials.

### Injection of Hydrogen Peroxide into *Drosophila*

Flies were injected with 1% hydrogen peroxide in PBS, and with PBS alone as a control. Green food coloring (Kroger brand) was added to the solutions to aid in liquid handling and scoring of injections. Microneedles were made using the PN-30 puller (Narishige) and Brosil glass capillary tubing (1.0 mm OD×0.75 mm ID; FHC Inc.). The needles were graduated before use with a scale of 1/32 inch, and ∼3 ul of solution was added to the needle. The needles with solution were then assembled into the FemtoJet express microinjector (Eppendorf). The flies were anaesthetized using CO_2_ and positioned on the pad with the abdomen oriented towards the needle using brushes. The colored solution was then injected into the abdomen of the adult fly using the microinjector; the volume injected was ∼0.05 ul per fly, based on the gradation markings on the needle.

### *Drosophila* Activity Monitor Assay

After the flies regained consciousness, the vials were placed into separate TriKinetics® *Drosophila* Population Monitors (DAM). These devices are each equipped with 3 rings that constantly emit an infrared beam through the vial. The interruptions of the beams, or activity units, caused by a fly passing through were then tallied in every hour by the DAM system. For the 4 day experiments (Figures 1, 2), flies were initially placed in control vials, and then at 21 hours the flies were transferred to control vials or vials adjusted to the indicated H_2_O_2_ or TBHP concentrations. For the 5-week experiments (Figure 4), flies were placed directly into food vials ±DOX. The vials were then monitored for the remainder of the experiments, with transfer to fresh vials every other day. The experiments were conducted in an incubator on a 12 h/12 h light/dark cycle at 25°C. A building power outage caused a several-hour gap in the data collection in one experiment (Figure 4). To allow further analysis of that time-course, the missing data were imputed using the theory of state space models with missing observations [60].

### Time Series Analysis of DAM Data

Seasonal time series like the fly activity data measured by the DAM consist of trend and periodic components. There are several approaches to analyzing such data [61]. The first is to extract the periodic and trend components of the data and analyze the residual. The second is to model the data as a whole. Due to the non-stationary nature of the fly activity data, the second technique was used, and an ARIMA (*p, d, q*)*_s_* process was employed. The acronym ARIMA stands for “Auto-Regressive Integrated Moving Average”, where p refers to the autoregressive, d the integrated, and q the moving average parts of the time series model. In [62] methods for choosing a suitable ARIMA model are discussed. A suitable ARIMA model can be selected using Akaike's Information Criterion (AIC), the model with the smallest AIC being chosen. Fitting can be performed using the function *arima* in the package *stats* in R [63]. The model used here included both differencing and a seasonal AR term of the form ARIMA ((1, 0, 1)x(1, 1, 1)_24_). The fit of the model was verified via both residual analysis, and the auto-correlation function of the residuals and the portmanteau lack-of-fit test (Ljung-Box test) [64]. Once the model was fitted to the data, statistics such as means and standard errors of the means were calculated.

### Multiple Camera 3D Tracking and DsRED Fluorescence Assay

Tracking of multiple fly locomotor activity was conducted using previously published methods [22], [32], [33]; detailed protocols are available for download from the laboratory website (http://towerlab.USC.edu/). All tracking experiments were done with young male flies (4-6 days old) and were initiated at the same time of day (4PM). Flies were placed in standard 25×75 mm polyethelene culture vials with food at the bottom, and stoppered with cotton at the top. The food was colored blue (Kroger brand food color) to facilitate tracking. The vials were placed in the center of a circular camera rig, 70 cm in diameter. Multiple calibrated and synchronized Flea digital cameras (Point Grey) were mounted on the camera rig, facing downward at a distance of 15 cm from the vials. Experiments involving only visible light tracking used three cameras [32], whereas all other experiments utilized six cameras [33]. Each camera was fitted with a 8 mm megapixel fixed focal lens (Edmund Optics). Tracking of gene expression using DsRED reporter transgenes in multiple flies was accomplished using published methods [33]. Briefly, the excitation light source was a 5W Luxeon V star 550 nm endura bright green lambertian LED (Optotech, Cat # OT16-5100-G). The LED was powered with a xitanium 700 mA LED driver (Optotech, AC converter Cat # OTMI-0060). 585 nm barrier filters (Edmund Optics, Cat # NT39-417) were placed between the sensors and the lenses of three cameras to detect DsRED expression. The fluorescence tracking assays were conducted in a dark room where the only source of illumination was the green LED. To track flies in alternating light/dark cycles, the light period was generated with a Luxeon III star white lambertian LED, and the dark period was illuminated using only a 5W Luxeon III star 630 nm endura red lambertian LED (Optotech, Cat # OT16-5100-R); the *Drosophila* photoreceptor pigments are not responsive to red light [65]. Prior to the tracking assays, flies were cultured to the indicated age by transfer to new food every other day, under a 12 hr/12 hr light/dark cycle. Activity is plotted as distance moved (cm) per hour.

### Fly Movement Parameters

To allow comparison of the effects of dietary hydrogen peroxide and SOD over-expression on fly movement behavior, the data from the tracking assay was used to calculate the following four parameters [66]:

#### Speed (cm/sec)

This is the distance traveled by a single fly in one second or 60 frames.

#### Average speed over an interval (cm/sec)

This is the average distance traveled by a fly over an interval of six seconds. This number was calculated by taking an interval width of±3 seconds around the current time instant.

Directional heading change (radians/sec). This is the angle between the two tangent vectors of the fly position in the 3D trajectory over a period of one second or 60 frames. The angle was calculated by taking the inverse cosine of the dot product of the two normalized vectors.

Directional heading change over an interval (radians/sec). This is the average directional heading change over an interval of six seconds. This number, like that of the Average Speed, was over an interval size of ±3 seconds around the current time instant. For the 48-hour plot of heading changes (Figure 2D), the value plotted is the change in heading of the fly in every second, averaged per hour.

### Measuring Time to Increased Activity

To determine the amount of time required for H_2_O_2_ to have an effect on the fly, five young (4–6 day old) adult male Oregon-R flies were placed in individual vials with food adjusted to 1.0% H_2_O_2_, and their activity was tracked for ∼30 minutes. We estimated the time from the first contact of each fly with the food until an increase in its activity was detected, where activity was measured as distance traveled in cm per hundredth of a minute. To do this it was assumed that each activity series had a changepoint at an unknown time, *t*. The series before and after time *t* were modeled as ARIMA(*p,d,q*) time series with (for simplicity) common values of *p, d* and *q*, but their own parameters. For each value of *t* the AIC from the two segments was found, and these values were plotted as a function of *t*; the value of *t* giving the smallest AIC was chosen as the breakpoint. This approach is essentially a simplified version of the method of Davis and coworkers, in which an *a priori* unknown number of breakpoints is estimated [67]. We chose ARMA(5,0,0) models, although the results were little influenced by other models we tested (data not shown). Supplemental Figure S3 shows the original series and the fitted means in each segment. The estimated breakpoints were 5.8, 7.98, 10.13 and 11.37 and 20.85 minutes (average = 11.23 minutes, SE 5.78 minutes). Each of the fitted models was compared to one with no breakpoint; the models with the breakpoint showed highly reduced AIC values.

## Supporting Information

Figure S1 Change in fly activity due to dietary hydrogen peroxide. Time series of activity data from the hydrogen peroxide (H_2_O_2_) dose-response in Figure 1A is presented. A seasonal-trend decomposition procedure decomposed the raw time-series activity data (gray) into a seasonal component indicating rhythms, and a trend component indicative of changes in overall activity (blue). This was performed in R using the function stl. A linear model was fit to the trend component of the raw activity time series (red) to demonstrate the trend of increasing activity for flies fed H_2_O_2_. Next, an ARIMA time series modeling approach (Material and methods) was utilized for further analysis which revealed average activity (with standard errors in parentheses) as follows: 0.1% H_2_O_2_ 36.2 (1.67); 0.5% H_2_O_2_ 43.7 (2.30); 1.0% H_2_O_2_ 71.1 (1.61); and 2.0% H_2_O_2_ 78.8 (2.41); Control 20.5 (1.20).
(3.37 MB TIF)

Figure S2 Effect of dietary TBHP on adult fly activity. (A) Oregon-R wild-type male flies fed 0.05% TBHP (red) or 0.1% TBHP (blue) and mock-fed controls (black), with data expressed as activity units per fly. For the TBHP experiments triplicate vials of 25 flies each were assayed and averaged for each condition. Drug treatment began at 21 hours. (B) The data are the same as in (A), and here are plotted to show difference in average activity per fly between experimental conditions and control.
(1.23 MB TIF)

Figure S3 Analysis of activity breakpoints. Activity time series for five young (4–6 days old) adult male Oregon-R flies from a tracking experiment to determine the amount of time required for H_2_O_2_ to have an effect on the fly. Flies were placed in individual vials with food adjusted to 1.0% H_2_O_2_, and their activity was tracked for approximately 30 minutes. Each panel corresponds to a separate fly; the red lines show the mean activity (distance traveled (cm per hundredth of a minute)) prior to and after the increase in activity.
(5.55 MB TIF)
