# Supplementary figures and images for: Hydrogen Peroxide Stimulates Activity and Alters Behavior in Drosophila melanogaster
Source: PLoS One. 2009 Oct 28;4(10):e7580. doi: 10.1371/journal.pone.0007580 (PMC2763216; doi:10.1371/journal.pone.0007580)

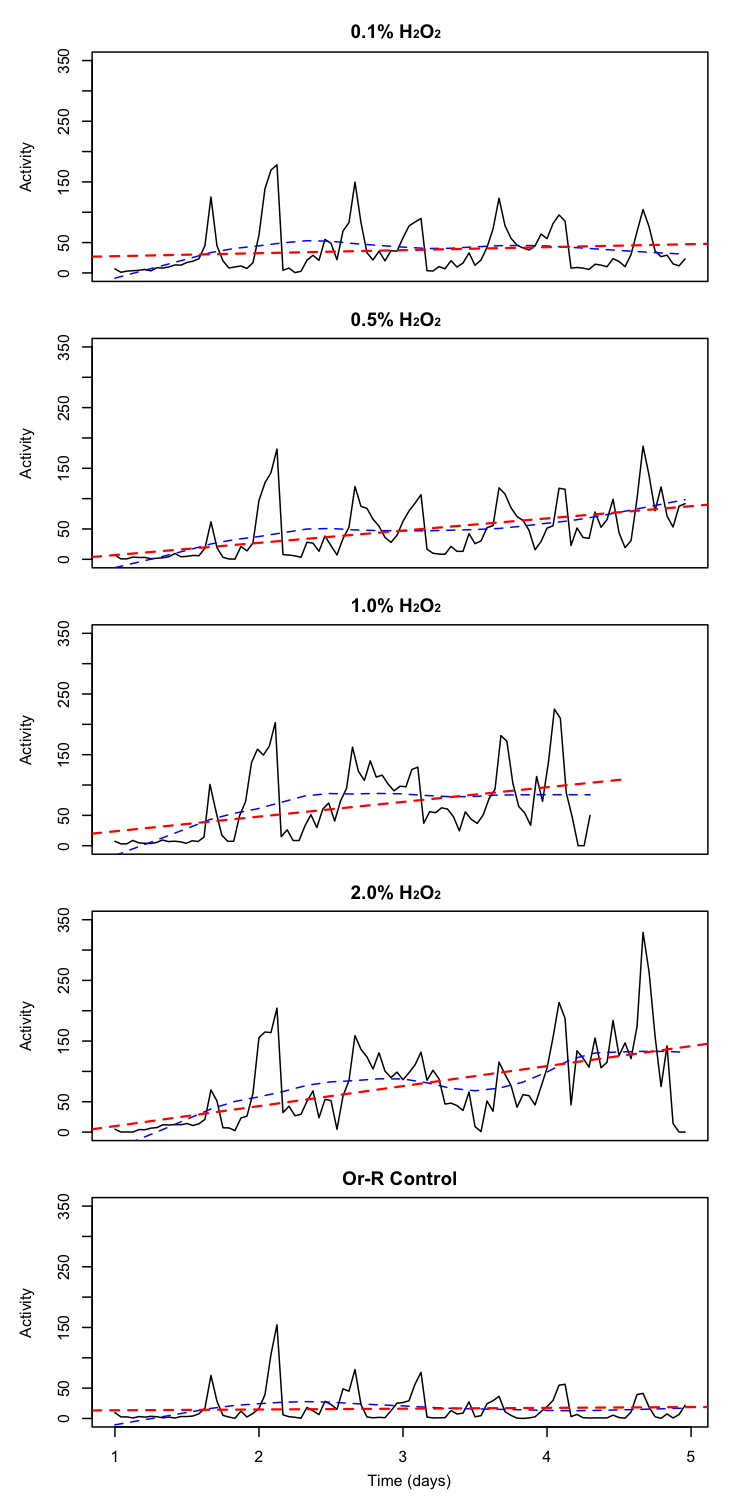

Supplement: Figure S1 — Change in fly activity due to dietary hydrogen peroxide. Time series of activity data from the hydrogen peroxide (H2O2) dose-response in Figure 1A is presented. A seasonal-trend decomposition procedure decomposed the raw time-series activity data (gray) into a seasonal component indicating rhythms, and a trend component indicative of changes in overall activity (blue). This was performed in R using the function stl. A linear model was fit to the trend component of the raw activity time series (red) to demonstrate the trend of increasing activity for flies fed H2O2. Next, an ARIMA time series modeling approach (Material and methods) was utilized for further analysis which revealed average activity (with standard errors in parentheses) as follows: 0.1% H2O2 36.2 (1.67); 0.5% H2O2 43.7 (2.30); 1.0% H2O2 71.1 (1.61); and 2.0% H2O2 78.8 (2.41); Control 20.5 (1.20). (3.37 MB TIF) [file pone.0007580.s001.tif]

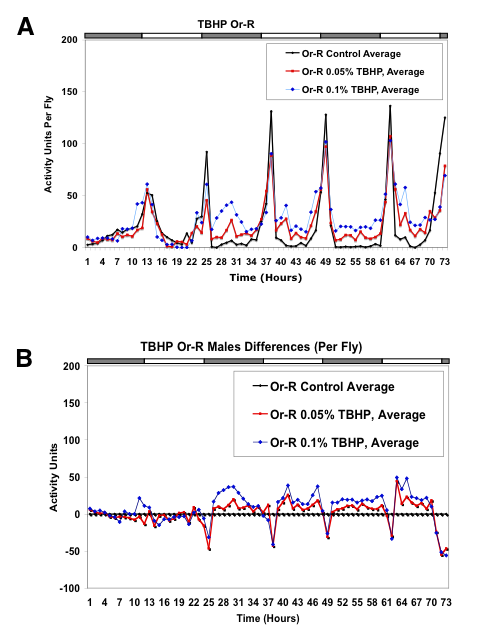

Supplement: Figure S2 — Effect of dietary TBHP on adult fly activity. (A) Oregon-R wild-type male flies fed 0.05% TBHP (red) or 0.1% TBHP (blue) and mock-fed controls (black), with data expressed as activity units per fly. For the TBHP experiments triplicate vials of 25 flies each were assayed and averaged for each condition. Drug treatment began at 21 hours. (B) The data are the same as in (A), and here are plotted to show difference in average activity per fly between experimental conditions and control. (1.23 MB TIF) [file pone.0007580.s002.tif]

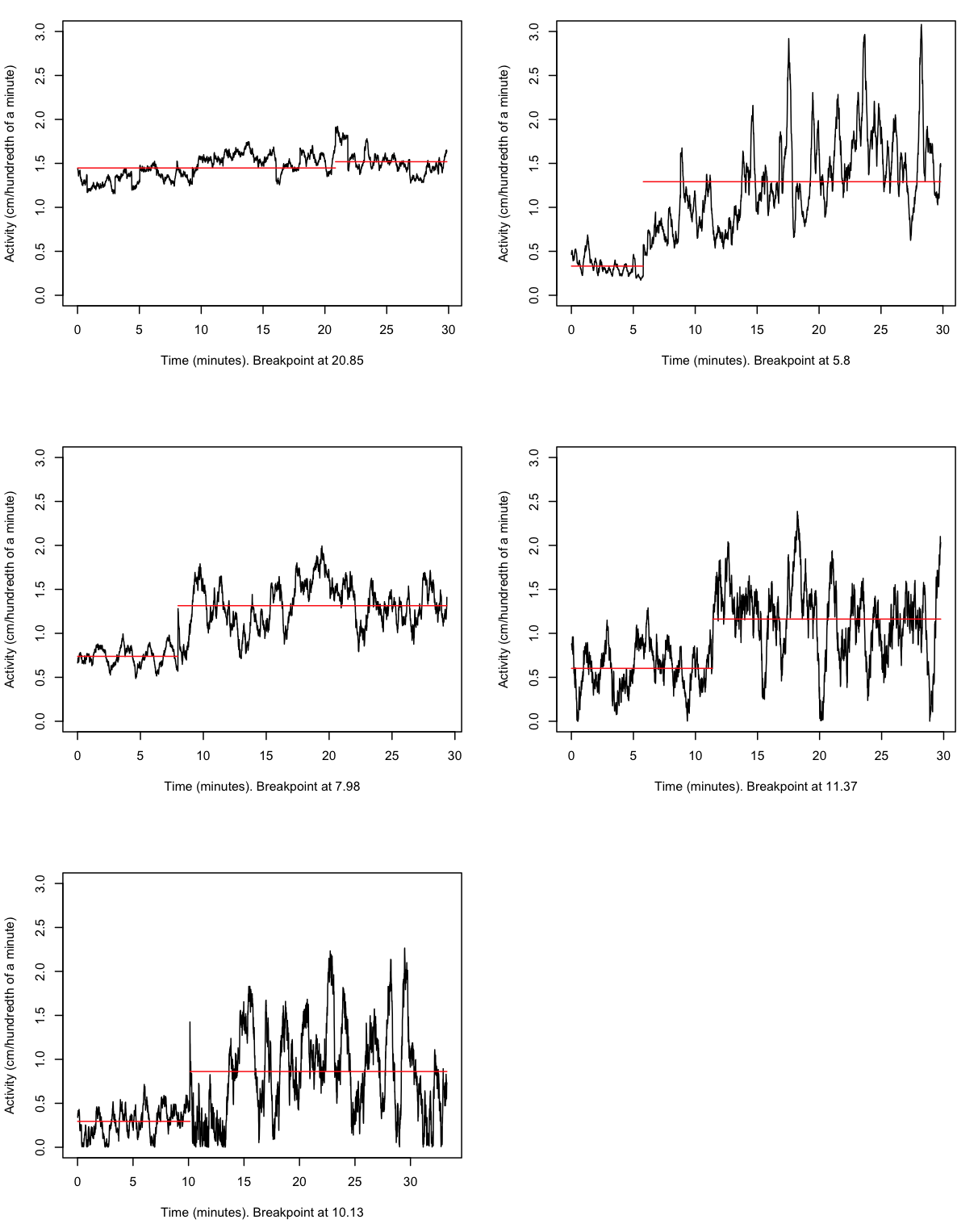

Supplement: Figure S3 — Analysis of activity breakpoints. Activity time series for five young (4–6 days old) adult male Oregon-R flies from a tracking experiment to determine the amount of time required for H2O2 to have an effect on the fly. Flies were placed in individual vials with food adjusted to 1.0% H2O2, and their activity was tracked for approximately 30 minutes. Each panel corresponds to a separate fly; the red lines show the mean activity (distance traveled (cm per hundredth of a minute)) prior to and after the increase in activity. (5.55 MB TIF) [file pone.0007580.s003.tif]
